# Differential Effects of 2-Deoxyglucose and Glucose Deprivation on 4-Hydroxynonenal Dependent Mitochondrial Dysfunction in Primary Neurons

**DOI:** 10.3389/fragi.2022.812810

**Published:** 2022-02-21

**Authors:** Matthew Dodson, Gloria A. Benavides, Victor Darley-Usmar, Jianhua Zhang

**Affiliations:** ^1^ Center for Free Radical Biology, University of Alabama at Birmingham, Birmingham, AL, United States; ^2^ Department of Pathology, University of Alabama at Birmingham, Birmingham, AL, United States; ^3^ Department of Veterans Affairs, Birmingham VA Medical Center, University of Alabama at Birmingham, Birmingham, AL, United States

**Keywords:** 2DG, HNE, mitochondria, bioenergetics, primary neurons, electron transport chain, FCCP, glucose

## Abstract

Mitochondrial dysfunction and metabolic decline are prevalent features of aging and age-related disorders, including neurodegeneration. Neurodegenerative diseases are associated with a progressive loss of metabolic homeostasis. This pathogenic decline in metabolism is the result of several factors, including decreased mitochondrial function, increased oxidative stress, inhibited autophagic flux, and altered metabolic substrate availability. One critical metabolite for maintaining neuronal function is glucose, which is utilized by the brain more than any other organ to meet its substantial metabolic demand. Enzymatic conversion of glucose into its downstream metabolites is critical for maintaining neuronal cell growth and overall metabolic homeostasis. Perturbation of glycolysis could significantly hinder neuronal metabolism by affecting key metabolic pathways. Here, we demonstrate that the glucose analogue 2-deoxyglucose (2DG) decreases cell viability, as well as both basal and maximal mitochondrial oxygen consumption in response to the neurotoxic lipid 4-hydroxynonenal (HNE), whereas glucose deprivation has a minimal effect. Furthermore, using a cell permeabilization assay we found that 2DG has a more pronounced effect on HNE-dependent inhibition of mitochondrial complex I and II than glucose deprivation. Importantly, these findings indicate that altered glucose utilization plays a critical role in dictating neuronal survival by regulating the mitochondrial response to electrophilic stress.

## Introduction

Oxidative stress is a prevalent feature of stroke and neurodegenerative diseases (Malkus et al., 2009; Redmann et al., 2018; Hill et al., 2019; Austad et al., 2021). As a major consequence of oxidative stress, accumulation of lipid peroxidation products and their adduction to target molecules is prevalent in the progression of these pathologies (Butterfield and Lauderback, 2002; Muralikrishna Adibhatla and Hatcher, 2006; Sultana et al., 2006; Ferretti et al., 2008; Butterfield et al., 2010). 4-hydroxynonenal (HNE) is a highly reactive lipid peroxidation product that forms protein adducts and stimulates the formation of other reactive species in stroke and neurodegenerative disease brains (Yoritaka et al., 1996; Reed et al., 2009; Dubinina and Dadali, 2010; Perluigi et al., 2012). Pathologically, HNE levels have been reported to be in the range of 1–10 μM in patient plasma, tissues, and neurons (Berg et al., 1997; Urabe et al., 2000; Wan-Chi Lee et al., 2012). Mechanistically, mitochondrial proteins are major targets of HNE, which has been shown to form adducts with respiratory chain subunits, increase reactive species production, and decrease mitochondrial reserve capacity, all of which contribute to bioenergetic dysfunction (Chen et al., 1998; Dranka et al., 2011; Schneider et al., 2011). Damaged mitochondria, which can generate secondary reactive oxygen species and amplify the initial oxidative insult, are removed by autophagy (Hill et al., 2012; Jisun Lee et al., 2012; Redmann et al., 2014). As autophagy is important in clearing damaged proteins and organelles, particularly during oxidative stress, changes in autophagic activity can modulate the effects of HNE on cellular bioenergetics and thereby impact cell death (Pan et al., 2008; Sarkar et al., 2009; Giordano et al., 2014). For example, we have shown in primary cortical neurons that HNE significantly alters the mitochondrial network and inhibits mitochondrial function, with the higher concentrations also suppressing autophagy (Dodson et al., 2017). These data are consistent with the hypothesis that autophagy and mitochondrial function are intimately linked.

Cellular bioenergetics, at the level of the mitochondrion, are closely linked to glucose metabolism through provision of pyruvate and maintenance of both the NADH/NAD+ and NADPH/NADP+ redox couples. The brain, like many other post-mitotic tissues with a high-energy demand, relies heavily on cellular metabolism and bioenergetics for normal function and survival. Energy utilization centers on ATP production mainly *via* mitochondrial oxidative phosphorylation or glycolysis, with alterations in ATP generation resulting in changes to normal metabolite production and use by the cell. Glycolysis is particularly important in brain cell metabolism, and as such the uptake and utilization of glucose is tightly controlled (Benarroch, 2014). Glucose-derived metabolites are critical for mitochondrial function, post-translational protein modifications, antioxidant defense, and nucleotide synthesis. Mitochondrial dysfunction is a key component of the pathological mechanisms underlying neurodegenerative disease and other syndromes with an energetic component. For example, mitochondrial complex I has been shown to be targeted by neurotoxins (including rotenone, MPTP, and 6-hydroxy-dopamine) that induce Parkinsonism in human patients and in animal models (Giordano et al., 2012). An accumulation of the mitochondrial complex II substrate succinate has been shown to impact reperfusion injury (Chouchani et al., 2014). We have previously shown that inhibition of glucose processing by hexokinase using the competitive inhibitor 2-deoxyglucose (2DG) suppresses autophagy and enhances HNE-induced cell death in differentiated SH-SY5Y cells (Dodson et al., 2013b). More recently, it has been shown that modulation of glucose metabolism also regulates the autophagy lysosomal pathway, which is responsible for clearance of damaged proteins and organelles under a variety of stress conditions (Dodson et al., 2013a).

There are 4 isoforms of hexokinase (I-IV), with hexokinase I representing the primary isoform found in the brain (Roberts and Miyamoto, 2015). Interestingly, outside of its primary function in metabolizing glucose, hexokinase has also been shown to regulate cell survival through interactions with a variety of protein binding partners. At the mitochondrial level, metabolic coupling of glycolysis to the mitochondrial respiratory chain occurs through a direct interaction between hexokinase I and II and the mitochondrial protein VDAC in the outer mitochondrial membrane (Pastorino and Hoek, 2008). Hexokinase binding to VDAC prevents it from interacting with pro-apoptotic Bcl family member proteins, such as Bax, which triggers mitochondrial outer membrane permeabilization (MOMP) and activation of apoptosis (Green and Kroemer, 2004). Recent studies have also shown that under hypoglycemic conditions, hexokinase II can interact with the autophagy regulator protein mechanistic target of rapamycin (mTOR), allowing for activation of the autophagy pathway (Roberts et al., 2014). Targeted transduction of hexokinase II into nigral dopaminergic neurons has also been shown to prevent 1-methyl-4-phenyl-1,2,3,6-tetrahydropyridine (MPTP) and rotenone-induced cell loss and motor deficits as a model of Parkinson’s disease (Corona et al., 2010). These studies indicate that hexokinase has the potential to act as a major regulator of neuronal cell viability by integrating metabolic function with autophagy and apoptosis, particularly at the level of the mitochondria.

To determine how altering glucose utilization affects neuronal mitochondria under basal and stressed conditions, we investigated the effect of inhibiting hexokinase using 2DG, or depriving neurons of glucose, on neuronal bioenergetics in the presence and absence of the PD-relevant electrophilic stressor HNE. Interestingly, we found that 2DG, but not glucose deprivation, significantly exacerbated mitochondrial dysfunction in the presence of HNE. Furthermore, 2DG altered HNE modification of specific mitochondrial complexes, as indicated by changes in oxygen consumption following the provision of different oxidizable substrates. These findings provide insight into the key role of glucose utilization in maintaining mitochondrial function and coupled with results from us and others indicating it affects autophagy, indicate that glucose metabolism could be a key mediator of PD onset and progression.

## Materials and Methods

### Materials

HNE (393204) was obtained from Calbiochem. Oligomycin (75351), FCCP (C2920), antimycin-A (A8674), rotenone (R8875), pyruvate (P5280), malate (M6413), ADP (A2754), succinate (S2378), and 2-deoxyglucose (2DG) (D8375) were obtained from Sigma. Neurobasal medium (21103-049), B-27 supplement (17504044-044), L-Glutamine (25030-081), and Penicillin/Streptomycin (15140-122) were obtained from Life Technologies. Trypan Blue Solution (25-900-CI) was obtained from CellGro.

### Cell Culture

Primary E18 rat cortical neurons were isolated, and cultured in Neurobasal medium containing 2% B27 supplement, 1% penicillin/streptomycin and 0.5 mM L-glutamine. All experiments used 7–10 days *in vitro* (DIV) cultures. DIV 7-10 neurons were treated in phenol free DMEM supplemented with XF media (1 mM pyruvate, 0 mM or 5 mM D-glucose, or 5 mM D-glucose + 20 mM 2DG) for 4 h in the presence or absence of 0, 5, 10, or 15 μM HNE. All animal procedures were approved by UAB institutional IACUC.

### Assessment of Cell Viability

Primary cortical neurons were plated at 80,000 cells per well in 96-well plates, and following treatment, trypsinized and resuspended in Neurobasal media. Trypan blue was added, and the cells non-permeable to trypan blue were counted as viable.

### Seahorse Extracellular Flux Assays

Primary cortical neurons were plated at 40,000 cells per well in Seahorse XFe-96 plates. Mitochondrial function was measured using the Seahorse XFe-96 analyzer. Basal oxygen consumption rate was determined, followed by injection with the following: 1 μg/ml oligomycin (O) to determine OCR linked to ATP production, 1 μM FCCP (F) to determine the maximal OCR, and 10 μM antimycin A (A) to determine non-mitochondrial OCR. All OCR measurements are represented as pmol oxygen consumed per minute.

### Mitochondrial Electron Transport Chain Activity Assay

The standard assay for mitochondrial function can be further optimized to indicate changes in the function of specific mitochondrial complexes. The mitochondrial membrane is permeabilized using 20 μg/ml of the detergent saponin, then specific complex I and II substrates [ADP + pyruvate/malate (10 mM/1 mM) and ADP + succinate (1 mM/10 mM), respectively] are provided. Substrate provision following permeabilization, coupled with the complex I inhibitor rotenone (1 μM), followed by treatment with the complex II inhibitor antimycin A (10 μM), allows for determination of specific changes in mitochondrial complex I or II following treatment. This assay, coupled with a similar assay for complex V function that includes saponin permeabilization, followed by addition of buffer containing FCCP (1 μM) and the same substrates, provides a detailed assessment of mitochondrial respiratory chain function and health in intact cells.

### Statistical Analysis

Data are reported as mean ± SEM. Graphed data represent technical replicates (*n* = 3–5 per group) from a single experiment that was repeated for validation. Comparisons between two groups were performed using unpaired Student’s *t-*tests. Comparisons among multiple groups were performed using a one-way analysis of variance (ANOVA), followed by post hoc Tukey’s HSD test. A *p*-value of ≤0.05 was considered statistically significant.

## Results

### 2DG Enhances HNE-Induced Cell Death to a Greater Extent Than Glucose Deprivation in Primary Rat Cortical Neurons

**FIGURE 1:**
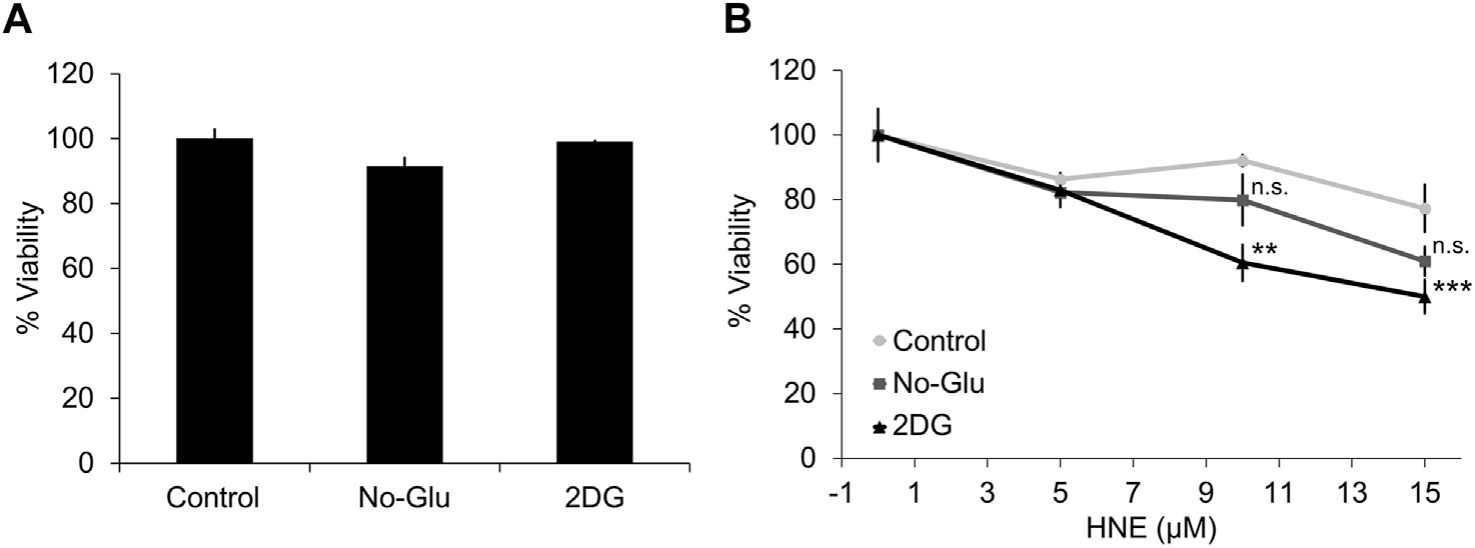
2DG, but not glucose deprivation, exacerbates HNE-induced cell death in primary cortical neurons. Rat E18 primary cortical neurons were treated on DIV7-10 with either 5 mM or 0 mM glucose, or 5 mM glucose + 20 mM 2-DG for 4 h in the presence or absence of 0, 5, 10, or 15 μM HNE. **(A)** Cell counts for neuronal viability following the indicated treatments in the absence of HNE. **(B)** Cell counts for neuronal viability following the indicated treatments in the presence of 0, 5, 10, or 15 μM HNE. **p < 0.001 and ***p < 0.0001 compared to 5 mM glucose group. Data = mean ± SEM, *n* = 3. One way ANOVA.

We have previously reported that 2DG, but not glucose deprivation, enhances HNE-induced apoptosis and cell death, and suppresses autophagy in differentiated SH-SY5Y cells (Dodson et al., 2013b). To test whether 2DG or glucose deprivation affect mitochondrial function and cell death, primary rat cortical neurons were treated with medium, plus 5 mM (control) or 0 mM glucose (No-Glu), 5 mM glucose + 20 mM 2DG (2DG) for 4 h in the presence or absence of 0–15 μM HNE. Neither 2DG, nor 0 mM glucose alone caused a significant decrease in cell viability (Figure 1A). However, in the presence of 10 and 15 μM HNE, 2DG caused a greater decrease in cell viability, ∼65% and 55% respectively, than glucose deprivation, ∼80% and 65%, compared to control media containing 5 mM glucose (Figure 1B).

### 2DG, but Not Glucose Deprivation, Exacerbates HNE-Induced Mitochondrial Dysfunction

**FIGURE 2:**
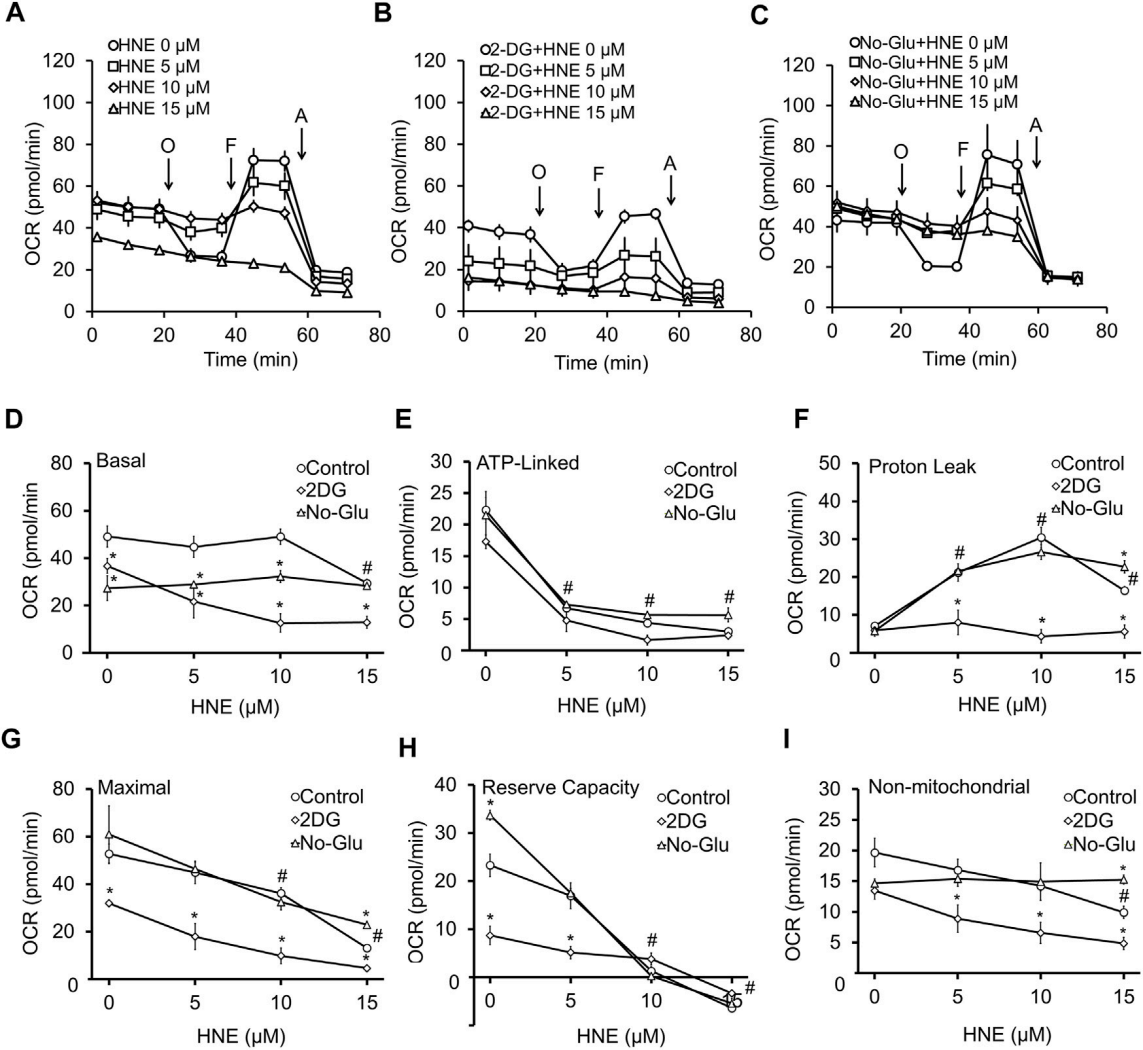
2DG, but not glucose deprivation, enhances HNE-induced mitochondrial dysfunction. DIV7-10 (E18) primary cortical neurons were treated with **(A)** 5 mM glucose (control), **(B)** 5 mM glucose + 20 mM 2DG or **(C)** 0 mM glucose in the presence or absence of 0, 5, 10, or 15 μM HNE for 4 h. O = 1 μg/ml oligomycin to determine OCR linked to ATP production, F = 10 μM FCCP to determine the maximal OCR, and A = 10 μM antimycin A to determine non-mitochondrial OCR. All OCR measurements are represented as pmol oxygen consumed per minute (pmol/min). Panel **(D–I)** represent a summary of basal OCR **(D)**, ATP-linked OCR **(E)**, Proton leak **(F)**, Maximal OCR **(G)**, Reserve capacity **(H)**, and non-mitochondrial OCR **(I)**. #*p* < 0.05 for the 5 mM glucose control at different HNE concentrations compared to 0 μM HNE, *p < 0.05 compared to the corresponding HNE concentration in 5 mM glucose group. Data = mean ± SEM, *n* = 4 or 5. Unpaired student’s t-test.

Next, we determined if neuronal mitochondrial function was affected by altered glucose utilization. To test this, we measured neuronal bioenergetic function with 5 mM glucose (Figure 2A), 5 mM glucose + 20 mM 2DG (Figure 2B), or 0 mM glucose (Figure 2C), in the presence of increasing concentrations of HNE. As we have previously reported (Dodson et al., 2017), in the control group (5 mM glucose) (Figure 2A), both 5 and 10 μM HNE had no effect, whereas 15 μM HNE significantly decreased basal OCR (Figure 2D). Additionally, a significant decrease in ATP-linked OCR, and a significant increase in proton leak was evident at 5 μM HNE (Figures 2E,F). Maximal OCR and reserve capacity were significantly decreased at 10 and 15 μM HNE (Figures 2G,H). The 15 μM HNE group was unresponsive to oligomycin and FCCP treatment. Non-mitochondrial OCR was decreased with 15 µM HNE (Figure 2I).

In media containing 5 mM glucose + 20 mM 2DG (Figure 2B), basal OCR was significantly lower than the 5 mM glucose control group (Figure 2A). 2DG significantly decreased basal OCR, maximal OCR, and reserve capacity at 0 μM HNE (Figures 2D,G,H), as well as significantly suppressed basal OCR, maximal OCR, and non-mitochondrial OCR, at 5–15 μM HNE (Figures 2D,G,I). Interestingly, 2DG prevented proton leak at 5–15 μM HNE (Figure 2F) when compared to the corresponding 5 mM glucose groups.

To determine if glucose deprivation had the same effect on HNE-induced mitochondrial dysfunction as 2DG, we next measured neuronal OCR following incubation with 0 mM glucose, in the presence of increasing concentrations of HNE (Figure 2C). Similar to 2DG, 0 mM glucose decreased basal OCR (Figure 2C) compared to the 5 mM glucose control (Figures 2A,D), either alone or in the presence of HNE; however, its effect on basal OCR was not as pronounced as 2DG, especially at the 10 and 15 μM concentrations of HNE (Figure 2D). ATP-linked OCR (Figure 2E), proton leak OCR (Figure 2F), maximal OCR (Figure 2G), reserve capacity (Figure 2H), and non-mitochondrial OCR (Figure 2I) in the 0 mM glucose group were very similar to the 5 mM glucose control up until 10 μM HNE. At 15 μM HNE there was a modest increase in proton leak (Figure 2F), maximal OCR (Figure 2G), and non-mitochondrial OCR (Figure 2I) (Figures 2E–I), and an increase of reserve capacity at 0 μM HNE (Figure 2H). These data indicate that glucose deprivation has less impact on HNE-induced mitochondrial dysfunction than 2DG.

### 2DG and Glucose Deprivation Alter HNE Targeting to Mitochondrial Complexes

**FIGURE 3:**
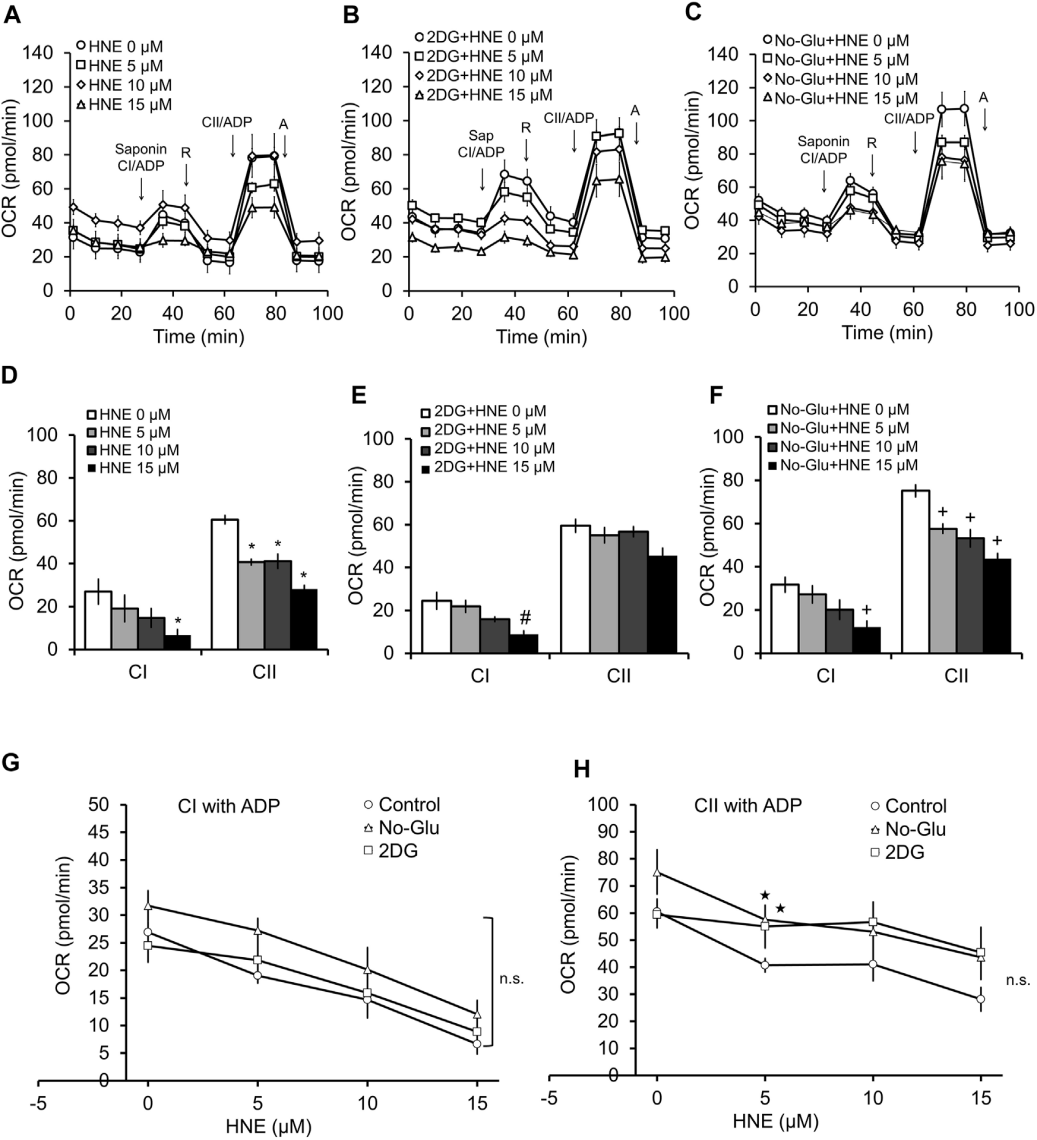
2DG and glucose deprivation have differential effects on complex V-controlled mitochondrial function in the presence of HNE. Rat primary cortical neurons were treated with 0, 5, 10, and 15 μM HNE in the presence of **(A)** 5 mM glucose, **(B)** 5 mM glucose + 20 mM 2DG, or **(C)** 0 mM glucose for 4 h. Sap = 20 μg/ml saponin, CI = Complex I, R = 1 μM rotenone, CII = Complex II, A = 10 μM antimycin A. First arrow indicates addition of ADP (1 mM) + complex I substrates malate (1 mM) and pyruvate (10 mM). Second arrow indicates addition of 1 μM rotenone to inhibit complex I function. The third arrow represents addition of ADP + the complex II substrate succinate (10 mM). The final arrow indicates addition of 10 μM antimycin A, which inhibits complex III function. OCR is represented in pmol/min. Panels **(D–F)** show the bar graphs calculated from panels **(A–C)**. Panels **(G,H)** show the line graphs of CI **(G)**, and CII **(H)**, substrate linked activity in response to increasing concentrations of HNE with glucose, with 2DG, or without glucose, calculated from **(A–C)**. *p < 0.05 compared to the 5 mM glucose control. #*p* < 0.05 compared to 0 μM HNE in 2DG group. +*p* < 0.05 compared to 0 μM HNE in no glucose group. ★*p* < 0.05 compared to corresponding HNE concentration in 5 mM glucose group. Data = mean ± SEM, *n* = 4 or 5. Unpaired student’s t-test.

We next used a permeabilization assay to determine if changing glucose availability alters HNE-induced damage of mitochondrial electron transport chain complexes I and II, which use pyruvate or the TCA cycle substrate succinate as substrates, respectively. Shown in Figures 3A–C are the profiles for the complex activity measurements. Similar to our previous study (Dodson et al., 2017), in the 5 mM glucose control conditions, HNE showed a concentration-dependent decrease in complex I linked substrate activity following the addition of ADP. A concentration-dependent decrease in complex II at all three HNE concentrations was also observed after addition of ADP + succinate (Figures 3A,D).

Interestingly, while in the presence of 2DG, complex I associated activity showed similar responsiveness to HNE as 5 mM glucose (Figures 3A,B,D,E,G), and increasing concentration of HNE no longer changed complex II associated activities (Figures 3A,B,D,E,H).

In contrast, glucose deprivation was associated with a trend in increased complex II activity across all concentrations of HNE compared to the 5 mM glucose control (Figures 3A,C,F,H), and a similar responsiveness to increasing concentrations of HNE was observed for both complex I and II substrate linked OCR compared to the 5 mM glucose control (Figures 3A,C,F,H). As shown in Figure 3G, complex I ADP-linked activities were similar across all groups, while Figure 3H indicates that there is a slight elevation of complex II function in the 5 µM HNE treated groups in the presence of either 2DG or glucose deprivation, compared to the 5 mM glucose group.

**FIGURE 4:**
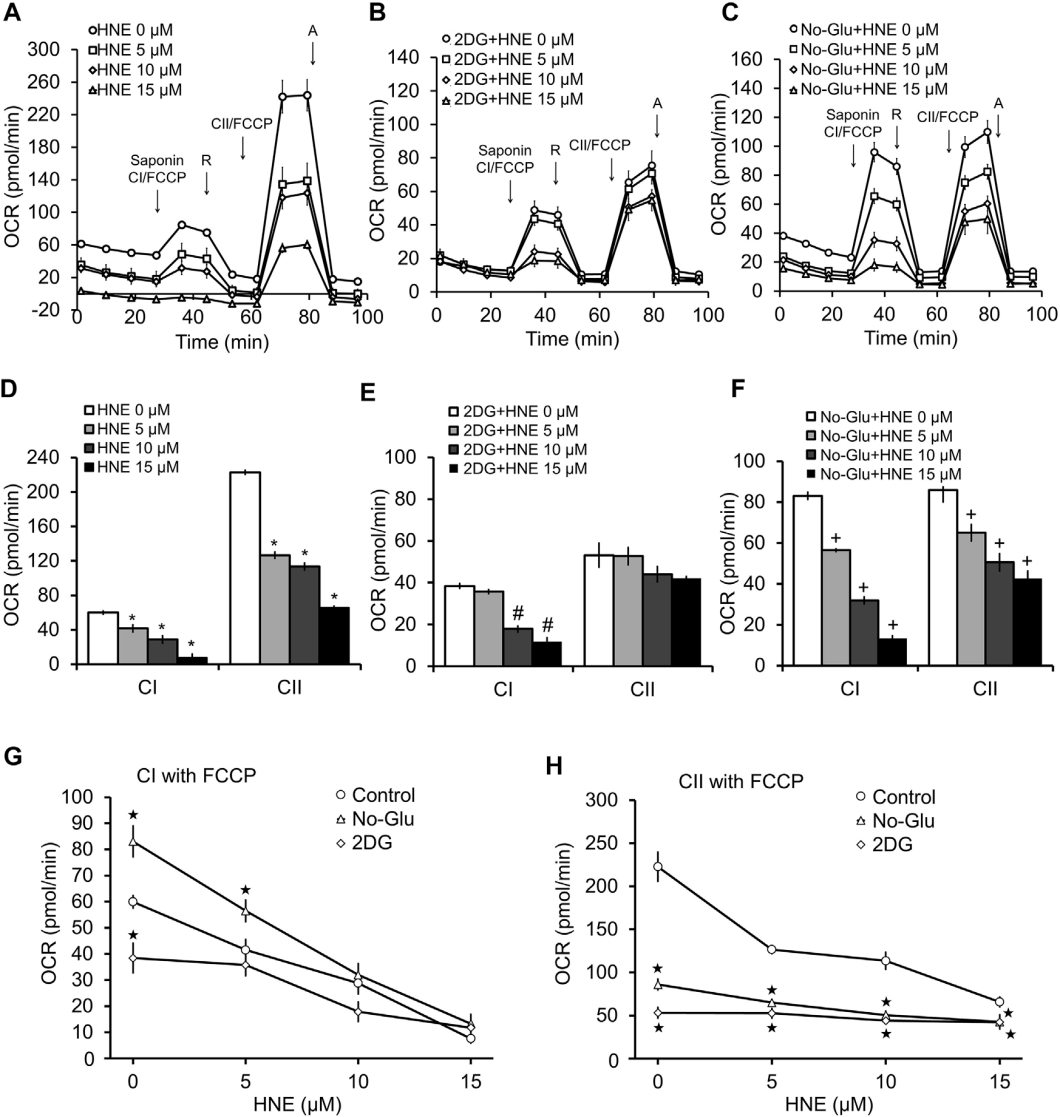
2DG and glucose deprivation have differential effects on complex V-independent mitochondrial function in the presence of HNE. Rat primary cortical neurons were treated the same as in Figure 3. **(A)** 5 mM glucose, **(B)** 5 mM glucose + 20 mM 2DG, or **(C)** 0 mM glucose for 4 h. Sap = 20 μg/ml saponin, CI = Complex I, R = 1 μM rotenone, CII = Complex II, A = 10 μM antimycin A. First arrow indicates addition of FCCP + complex I substrates malate (1 mM) and pyruvate (10 mM). Second arrow indicates addition of 1 μM rotenone to inhibit complex I function. The third arrow represents addition of FCCP (1 μM) + the complex II substrate succinate (10 mM). The final arrow indicates addition of 10 μM antimycin A, which inhibits complex III function. OCR is represented in pmol/min. Panels **(D,E)** show the bar graphs calculated from panels **(A–C)**. *p < 0.05 compared to 0 μM HNE for complex I substrate linked activity (CI), #*p* < 0.05 compared to 0 μM HNE for complex II substrate linked activity (CII). Panels **(G,H)** show the line graphs of CI **(G)**, and CII **(H)**, substrate linked activity in response to increasing concentrations of HNE with glucose, with 2DG and without glucose, calculated from **(A–C)**. *p < 0.05 compared to the 5 mM glucose control. #*p* < 0.05 compared to 0 μM HNE in 2DG group. +*p* < 0.05 compared to 0 μM HNE in no glucose group. ★*p* < 0.05 compared to corresponding HNE concentration in 5 mM glucose group. Data = mean ± SEM, *n* = 4 or 5. Unpaired student’s t-test.

Since, in coupled mitochondria, a decrease in complex I and II activity in the presence of ADP could be due to inhibition of complex V, we performed the same permeabilization assay in the presence of the uncoupler FCCP (1 μM) (Figure 4). FCCP eliminates control of oxygen consumption by uncoupling the proton gradient from ATP synthesis; thus, any changes in OCR observed are independent of complex V function. As expected for uncoupled mitochondria in the permeabilized cells, complex I activity is higher in the presence of FCCP than in the presence of ADP (Figures 3, 4). In the case of complex II linked uncoupled OCR, both 0 mM glucose and 2DG significantly decreased complex II substrate linked OCR relative to 5 mM glucose (Figure 4H). Compared to coupled mitochondria (Figure 3), complex I linked substrate activities were more sensitive to HNE, in the presence of FCCP (Figures 4A,D). Similar to the coupled mitochondria (Figure 3), complex II substrate linked respiration is no longer sensitive to HNE in 2DG treated cells (Figures 4B,E). In contrast, glucose deprivation (No-Glu) did not change sensitivity to HNE for these complexes compared to the 5 mM control, (Figures 4D,F). In the absence of HNE and in the presence of FCCP, 0 mM glucose increased, whereas 2DG decreased, complex I substrate linked OCR relative to 5 mM glucose (Figure 4G).

## Discussion

Metabolic decline occurs in the brain during normal aging and can be further enhanced by the development and progression of neurodegenerative diseases. Mitochondrial dysfunction, which is associated with increased oxidative stress, is a major driving force of the progression of Alzheimer’s, Parkinson’s, and Huntington’s disease, as well as several other neurological disorders (Lin and Beal, 2006; Austad et al., 2021). The progressive loss of glucose metabolism is also a prevalent feature of neurodegenerative disease, as indirectly shown by FDG-PET and MRI imaging, as well as measurement of concentrations of lactate, pyruvate, and TCA cycle metabolites in patient cerebrospinal fluid (Parnetti et al., 1995; Redjems-Bennani et al., 1998; Buckner et al., 2005; Edison et al., 2007; Schroeter et al., 2009; La Joie et al., 2012; Liguori et al., 2016; Marchitelli et al., 2018; Chen et al., 2021). Therefore, a better understanding of the metabolic dysfunction that occurs in the brain during neurodegenerative disease progression is needed, at both the mitochondrial level, as well as upstream regarding the use of major metabolites such as glucose.

Here, we tested the effects of blocking glucose availability and utilization on cellular bioenergetics in rat cortical neurons in the presence and absence of the reactive species HNE. HNE is a pathologically relevant reactive species that accumulates in Alzheimer’s and Parkinson’s disease brains, and we have shown previously that perturbation of glucose metabolism can greatly alter HNE-induced toxicity in differentiated SH-SY5Y cells (Yoritaka et al., 1996; Sayre et al., 1997; Dodson et al., 2013b). In rat cortical neurons, in the presence of physiological glucose concentrations, HNE caused a concentration-dependent decrease in neuronal viability, which was associated with increased mitochondrial proton leak, and decreased maximal mitochondrial respiration. These alterations to mitochondrial function were associated with a decrease in the mitochondrial reserve capacity, indicating that HNE specifically inhibits mitochondrial function in a concentration-dependent manner and decreases overall neuronal bioenergetic efficiency (Figures 1, 2).

Upon exposure to 2DG, cell viability, as well as both basal and maximal mitochondrial oxygen consumption were significantly decreased in the presence of HNE compared to the 5 mM glucose control. Interestingly, HNE effects on proton leak were lost in the presence of 2DG, possibly due to the already significant loss of basal respiratory function. We next exposed neurons to increasing concentrations of HNE in the presence of 0 mM glucose to determine if glucose deprivation had the same effect has 2DG treatment. In contrast to 2DG, glucose deprivation did not have as great an effect on HNE-induced cell death compared to control. Glucose deprivation effects on HNE-induced mitochondrial dysfunction were also similar to those observed in the normal glucose control.

Using a cell permeabilization assay, in the presence of either ADP (Figure 3) or FCCP (Figure 4), we found that 2DG’s effects on mitochondrial function are different from glucose deprivation, in that complex II substrate linked respiration is no longer responsive to HNE. It is important to note that some of the observed decreases in mitochondrial functionality caused by 2DG could be a result of the increased toxicity observed; however, the significant difference in the various OCR parameters, particularly at the higher concentrations of HNE, indicate that mitochondrial function in the surviving neurons is severely compromised. This finding demonstrates that perturbation of glucose metabolism could play a key role in dictating neuronal mitochondrial function and survival during stress, not only through substrate provision, but also by altering the targeting of reactive species to specific mitochondrial complexes. This also indicates that an inability to properly metabolize glucose could be a key driver of PD progression, particularly in the context of linking metabolic and autophagic dysfunction to neuronal cell death.

These findings show that specific perturbation of glucose utilization, not simple deprivation of neurons of their glucose supply, has detrimental effects on HNE-induced mitochondrial dysfunction. This could be due to 1) Phosphorylation of 2DG further decreasing cellular ATP, inhibiting the pentose phosphate pathway, affecting glycosylation, or enhancing ER stress (Laussel and Leon, 2020), 2) Inhibitory effects on autophagy resulting in accumulation of dysfunctional mitochondria and increased oxidative stress, or 3) Decreased hexokinase translocation to the mitochondria. It is also important to note that HNE could be modifying and inhibiting the function of other metabolite processing enzymes downstream of hexokinase, such as pyruvate dehydrogenase, which could also affect mitochondrial function in the presence of 2DG. A more detailed assessment of these possibilities will be performed in future studies. As noted above, a caveat of this study is that we have used 2DG as a competitive inhibitor of hexokinase. In the course of inhibition, it is phosphorylated, and this phosphorylated metabolite may also have downstream effects on the pentose phosphate pathway (Laussel and Leon, 2020). It is then possible that not all the effects we have observed with 2DG are directly attributable to hexokinase inhibition. However, in support of a major role for hexokinase, studies have shown that knockout/knockdown of this enzyme increases susceptibility to cell death, and it would then be likely that it would enhance the toxicity of stressors such as 4-HNE (Gimenez-Cassina et al., 2009; Mergenthaler et al., 2012; Lauterwasser et al., 2021). It is also important to note that 2DG has been considered as a cancer therapeutic, and as such understanding that it enhances HNE-dependent toxicity may be an important factor to consider in its clinical applications (Zhang et al., 2014). Taken together, these results demonstrate that dysfunctional glucose utilization may contribute to the metabolic decline, increased oxidative stress, and impaired protein turnover that drive neurodegenerative diseases, and that glucose metabolism may impact neuronal survival during stress.

## Data Availability

The raw data supporting the conclusion of this article will be made available by the authors, without undue reservation.
